# Congestive Heart Failure Exhibited Higher BMI With Lower Energy Intake and Lower Physical Activity Level: Data From the National Health and Examination Nutrition Survey

**DOI:** 10.3389/fcvm.2021.680371

**Published:** 2021-06-09

**Authors:** Tianyu Xu, Haobin Zhou, Zhuang Ma, Hao Zhang, Qingchun Zeng, Dingli Xu, Yuhui Zhang, Jian Zhang

**Affiliations:** ^1^State Key Laboratory of Cardiovascular Disease, Heart Failure Center, National Center for Cardiovascular Disease, Fuwai Hospital, Chinese Academy of Medical Sciences and Peking Union Medical College, Beijing, China; ^2^State Key Laboratory of Organ Failure Research, Department of Cardiology, Nanfang Hospital, Southern Medical University, Guangzhou, China

**Keywords:** congestive heart failure, body mass index, total nutrient intakes, physical activity, hemodilution, obesity

## Abstract

**Background:** Despite that nutritional deficiency existed in congestive heart failure (CHF), there is a large amount of CHF patients suffering from obesity. This study aimed to identify the differences for increased BMI or obesity in CHF patients.

**Methods:** This cross-sectional study included adults from the National Health and Nutrition Examination Survey 2007–2016. Differences were compared between CHF participants vs. non-CHF participants, and BMI ≥ 30 kg/m^2^ vs. BMI < 30 kg/m^2^ CHF participants.

**Results:** CHF participants were with higher BMI, lower energy and macronutrient intake, lower physical activity level and longer rest time, and lower hematocrit and hemoglobin level (all *P* < 0.05) than non-CHF participants. The prevalence of BMI ≥ 30 kg/m^2^ in participants with CHF was 53.48%. There was no significant difference observed in energy and macronutrient intake between CHF participants with BMI ≥ 30 kg/m^2^ or <30 kg/m^2^. The water intake (*P* = 0.032), sedentary time (*P* = 0.002), and hematocrit (*P* = 0.028) were significantly different between CHF with BMI ≥ 30 kg/m^2^ and with <30 kg/m^2^.

**Conclusion:** Compared with non-CHF participants, CHF participants exhibited higher BMI with lower energy and macronutrient intake, lower physical activity level, longer rest time, and hemodilution with lower hematocrit and hemoglobin level. Among CHF participants with BMI ≥ 30 kg/m^2^, higher sedentary time and hematocrit were observed.

## Highlights

- CHF participants were with higher BMI, lower energy and macronutrient intake, lower physical activity level and longer rest time, and lower hematocrit and hemoglobin level.
- Only water intake, sedentary time, and hematocrit were significantly different between CHF with BMI ≥ 30 kg/m^2^ and with <30 kg/m^2^.
- Longer resting time and an unbalanced diet may be associated with a higher prevalence of increased BMI or obesity in CHF participants.

## Introduction

Congestive heart failure (CHF) is a complex clinical condition that represents the final evolution of all cardiac diseases and a global public health problem which affects an estimated 26 million worldwide (1), which has a tremendous economic impact on the public healthcare system (2). Studies revealed that CHF is associated with alterations in cardiac energy metabolism (3) and that nutrition intake may influence the evolution of the disease progress (4). A deficiency in energy and macronutrient intake among CHF patients was reported, which caused an undernourished or malnutritional condition (5–7). Obesity [body mass index (BMI) ≥30 kg/m^2^], with up to 40% prevalence among CHF patients (8), is recognized as a major independent risk factor for the development of CHF (9) and has a paradoxical impact on the prognosis of CHF (10, 11). Studies indicated a non-linear U-shaped association between BMI and the risk of HF mortality, with a greater risk from being at the lowest group (mean BMI = 19.43 kg/m^2^), rather than being at the top category (mean BMI = 30.16 kg/m^2^) (12). The beneficial effects of weight loss in HF patients are still controversial, given the view that despite nutritional deficiency existed in CHF, there is a large amount of CHF patients suffering from obesity, and we still lacked studies to explain the reasons for the increased BMI in CHF patients. In this research, we used the data from the National Health and Examination Nutrition Survey (NHANES) 2007–2016 to identify the differences for increased BMI or obesity in CHF participants.

## Method

### Dataset

The NHANES data, a nationally representative multistage cross-sectional survey of the non-military and non-institutionalized population of the United States, was used as the data source (13). The data is released by the National Center for Health Statistics once every 2 years. Each survey cycle collects self-reported and directly measured information from participants who undergo a series of questionnaires in a detailed in-home interview such as health conditions, behaviors, and dietary intake, and a physical examination during mobile examination. Also, each participant provides their blood for laboratory tests. This survey included NHANES data from 2007 to 2016.

### Study Population

To identify the study population, we implemented the following exclusion criteria: participants <18 years old, participants who were pregnant or breastfeeding at the time of the survey, and participants who lack information on CHF diagnosis, dietary data, examination data, laboratory data, or questionnaire data. The final sample for this study consisted of 660 CHF participants and 20,923 non-CHF participants.

### Definition of CHF and Obesity

CHF was self-reported and was obtained from the medical condition files that were administered in the home by an interviewer using the Computer-Assisted Personal Interviewing system as part of the survey participant household interview. The final sample provided yes/no responses to the following question: “Has a doctor or other health professional ever told {you/SP} that {you/s/he}… had congestive HF?”

Height and weight were measured at the Mobile Examination Center (MEC) examination. BMI was calculated as weight in kilograms divided by the square of height in meters. BMI was analyzed as both a continuous variable and as a categorical variable dichotomizing into BMI ≥ 30 kg/m^2^ (obese) and BMI < 30 kg/m^2^ (non-obese) (14).

### Sample Demographics, Dietary Condition, and Other Covariates

We obtained information on age and sex from the NHANES self-reported demographics data. Blood pressures and heart rate (resting) were measured in the MEC examination by a physician. Averages of up to four values for systolic and diastolic blood pressures were recorded. Intakes of energy, water, and macronutrients, which consisted of protein, carbohydrate, total sugars, total fat, and cholesterol, were obtained from self-reported dietary data, and an average of 2 days was recorded. Physical activity, including work, and recreational activities, was classified as vigorous, moderate, bicycling or walking, and sedentary and was obtained by self-reporting the number of days they engaged in that activity in a typical week and the average duration they engaged in that activity. For each activity, the Metabolic Equivalent of Task (MET)-hr-week was calculated by multiplying the number of days, the mean duration, and the respective MET level (MET-hr-week = days * duration * MET level). The MET levels for each activity are provided as vigorous work/recreational-related activity = 8 MET, moderate work/recreational-related activity = 4 MET, and walking or bicycling for transportation = 4 MET. It was analyzed as a continuous variable for total activates MET and MET in each activity. Hematocrit (%) and hemoglobin (g/dL), which may imply hemodilution (15), were acquired from blood collected during the MEC examination using methods described by the US Department of Health and Human Services.

### Statistical Analysis

Data are expressed as the mean ± standard deviation or as the number (%). Differences between groups were tested by the chi-square test for categorical variables and independent Student's *t*-test, as well as ANOVA with Tukey's *post-hoc* analysis for continuous variables, as appropriate. Since the sample size is large, a simple application of the Lyapunov or Lindeberg's Central Limit Theorem guarantees large sample convergence of the weighted mean to a standard normal distribution, ensuring that the t-statistic would have a limiting t-distribution. We also used restricted cubic splines with three knots at the 5th, 25th, 50th, 75th, and 95th centiles to flexibly model the association of sedimentary time with BMI ≥ 30 kg/m^2^ in CHF participants and non-CHF participants, respectively. We tested for potential non-linearity by using a likelihood ratio test comparing the model with only a linear term against the model with linear and cubic spline terms. A *P* value < 0.05 was considered statistically significant. All tests were two-sided. All analyses were performed using R: A Language and Environment for Statistical Computing, version 3.1.3 (R Foundation for Statistical Computing, Vienna, Austria).

## Results

### Participants' Characteristics Between CHF and Non-CHF

**Figure 1:**
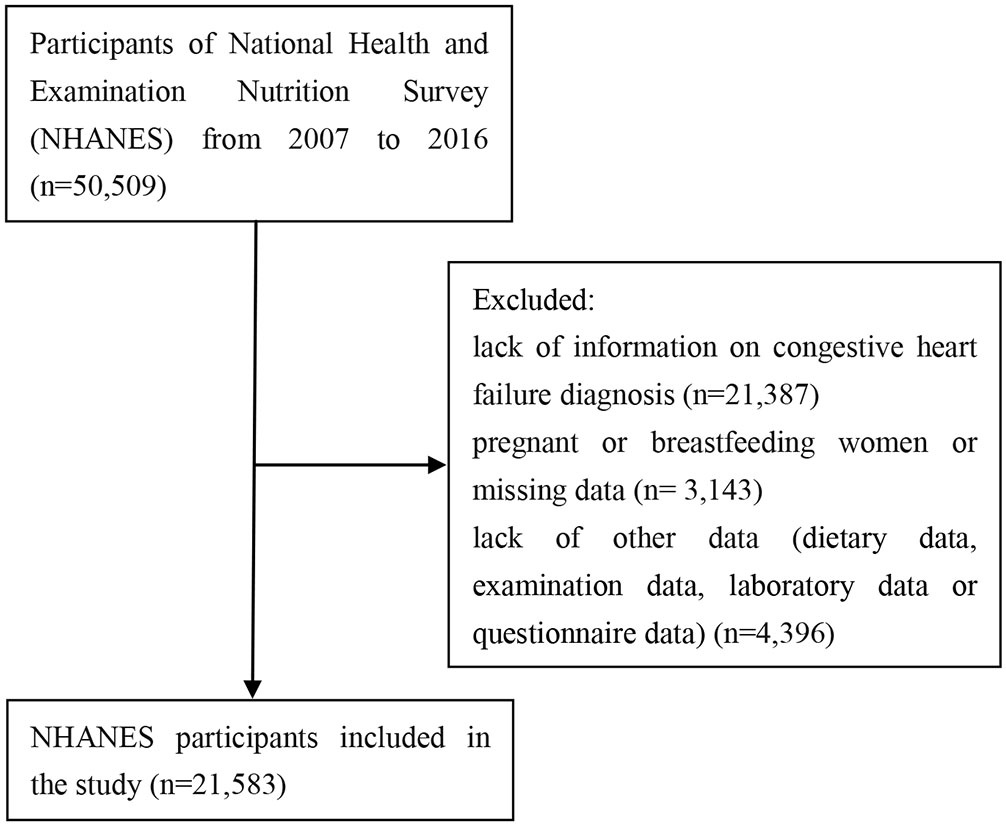
Flowchart for participant selection.

The flowchart for participant selection is outlined in Figure 1. Table 1 presents the clinical characteristics of CHF and non-CHF participants in NHANES 2007–2016. As noted, CHF participants were older than non-CHF participants (*P* < 0.05) with higher systolic blood pressure, lower diastolic blood pressure, and lower heart rate (all *P* < 0.05). Intake of total nutrients, including energy, protein, total carbohydrate, total sugar, total fat, and cholesterol, as well as water was much lower compared with that in non-CHF participants (all *P* < 0.05). Also, CHF participants had lower physical activity level (*P* < 0.05) and longer sedentary time (*P* < 0.05). Moreover, hemoglobin and hematocrit were lower in CHF participants (*P* < 0.05). However, BMI was higher in CHF participants than counterparts (*P* < 0.05).

**Table 1:** Characteristics of congestive heart failure group and non-congestive heart failure participants.

	**Congestive heart**	**Non-congestive heart**	***P-*value**
	**failure (*n* = 660)**	**failure (*n* = 20,923)**	
**Demographic and weight characteristics, mean (SD)**			
Age (y)	66.46 (12.72)	48.65 (17.51)	<0.001
**Sex**			
Male (no., %)	360 (54.55)	10,153 (48.53)	<0.001
BMI (kg/m^2^)	31.96 (8.54)	28.99 (6.80)	<0.001
SBP (mmHg)	129.82 (21.94)	123.26 (18.17)	<0.001
DBP (mmHg)	65.57 (15.03)	70.03 (12.76)	<0.001
Heart rate (beats/min)	71.02 (12.69)	72.81 (12.15)	<0.001
**Total nutrient intake, mean (SD)**			
Energy (kcal)	1734.71 (717.33)	2051.32 (872.86)	<0.001
Protein (gm)	69.85 (31.39)	80.65 (36.92)	<0.001
Carbohydrate (gm)	213.08 (94.19)	251.13 (111.62)	<0.001
Total sugars (gm)	93.56 (57.98)	110.98 (68.26)	<0.001
Total fat (gm)	66.36 (32.80)	76.58 (39.85)	<0.001
Cholesterol (mg)	266.10 (175.92)	286.80 (197.44)	0.0102
Water (gm)	531.16 (584.86)	646.00 (647.87)	<0.001
**Physical activity, mean (SD)**			
Total activity (MET-h/week)	24.54 (66.46)	57.68 (98.97)	<0.001
Vigorous activity (MET-h/week)	9.62 (42.83)	27.83 (70.54)	<0.001
Moderate activity (MET-h/week)	11.89 (28.80)	23.64 (42.33)	<0.001
Sedimentary activity (h/week)	6.81 (3.52)	5.79(3.39)	<0.001
Walk or bicycle (MET-h/week)	3.03 (14.95)	6.21 (20.27)	<0.001
**Complete blood count—whole blood, mean (SD)**			
Hemoglobin (g/dL)	13.48 (1.73)	14.08 (1.53)	<0.001
Hematocrit (%)	39.81 (4.82)	41.25 (4.24)	<0.001

*BMI, body mass index; SBP, systolic blood pressure; DBP, diastolic blood pressure; MET, metabolic equivalent of task; SD, standard deviation*.

### Participants' Characteristics by BMI Category

**Figure 2:**
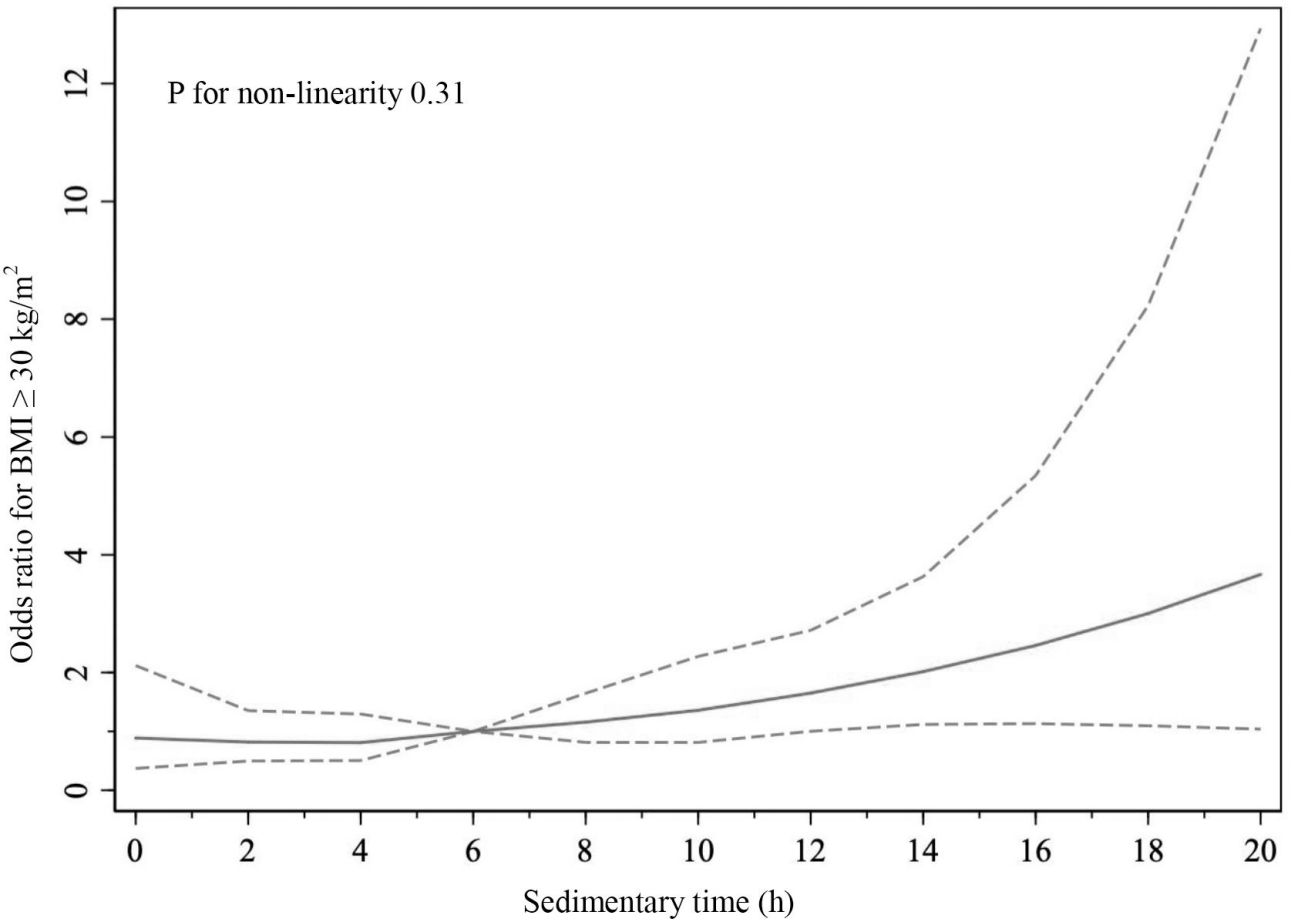
Association of sedimentary time with BMI ≥ 30 kg/m^2^ in CHF participants.

As shown in Table 2, the proportions of BMI ≥ 30 kg/m^2^ were 53.48% (*n* = 353) in CHF participants and 37.01% (*n* = 7,744) in non-CHF participants. Compared with BMI < 30 kg/m^2^ CHF participants, BMI ≥ 30 kg/m^2^ CHF participants showed significantly younger age, larger amount of water intake, longer sedentary time, and higher hematocrit (all *P* < 0.05). Among non-CHF participants, those with BMI ≥ 30 kg/m^2^ were older and had higher blood pressure and heart rate and lower energy, protein, total carbohydrate, and total sugar intake but higher cholesterol and water intake (all *P* < 0.05). Physical activity level was lower and sedentary time was longer (both *P* < 0.05) in BMI ≥ 30 kg/m^2^ non-CHF participants. Meanwhile, hemoglobin and hematocrit were lower in BMI ≥ 30 kg/m^2^ non-CHF participants than BMI < 30 kg/m^2^ ones (both *P* < 0.05). Restricted cubic spline showed that the risk of BMI ≥ 30 kg/m^2^ in CHF participants was increased with the sedimentary time (odds ratio 1.08, 95% confidence interval 1.03–1.13) (Figure 2), and such result could be also seen in participants without CHF (Supplementary Figure 1).

**Table 2:** Characteristics of congestive heart failure group and non-congestive heart failure group (stratified by obese and non-obese).

**BMI (kg/m^**2**^)**	**Congestive heart failure**			**Non-congestive heart failure**			^*^P-value
	**BMI < 30 kg/m^**2**^**	**BMI ≥ 30 kg/m^**2**^**	***P***	**BMI < 30 kg/m^**2**^**	**BMI ≥ 30 kg/m^**2**^**	***P***	
	**(*n* = 307)**	**(*n* = 353)**	**value**	**(*n* = 13,179)**	**(*n* = 7,744)**	**value**	
**Demographic and weight characteristics, mean (SD)**							
Age (y)	68.67 (12.86)	64.54 (12.30)	0.012	48.28 (18.17)	49.29 (16.30)	<0.001	<0.001
**Sex**							
Male (no., %)	178 (57.98)	181 (51.27)	<0.001	6,786 (51.49)	3,367 (43.48)	<0.001	<0.001
BMI (kg/m^2^)	25.34 (3.03)	37.72 (7.56)	<0.001	24.95 (3.13)	35.86 (5.78)	<0.001	<0.001
SBP pressure (mmHg)	130.38 (23.38)	129.34 (20.63)	0.89	122.01 (18.53)	125.40 (17.33)	<0.001	<0.001
DBP (mmHg)	64.13 (14.56)	66.81 (15.33)	0.04	69.15 (12.45)	71.51 (13.16)	<0.001	<0.001
Heart rate (beats/min)	70.05 (12.37)	71.84 (12.92)	0.24	71.73 (11.9)	74.64 (12.23)	<0.001	<0.001
**Total nutrient intakes, mean (SD)**							
Energy (kcal)	1759.97 (716.89)	1712.74 (718.01)	0.90	2079.46 (894.56)	2003.43 (832.49)	<0.001	<0.001
Protein (gm)	70.93 (32.74)	68.91 (30.19)	0.90	81.30 (37.76)	79.55 (35.44)	0.005	<0.001
Carbohydrate (gm)	217.22 (95.26)	209.48 (93.24)	0.81	256.08 (114.39)	242.69 (106.21)	<0.001	<0.001
Total sugars (gm)	96.15 (59.90)	91.31 (56.23)	0.80	112.65 (70.08)	108.13 (64.96)	<0.001	<0.001
Total fat (gm)	65.65 (30.90)	66.98 (34.41)	0.97	76.48 (40.51)	76.73 (38.72)	0.97	<0.001
Cholesterol (mg)	261.71 (181.87)	269.92 (170.75)	0.95	282.87 (200.12)	293.50 (192.62)	<0.001	<0.001
Water (gm)	457.69 (484.71)	595.06 (653.72)	0.032	615.04 (621.65)	698.70 (687.04)	<0.001	<0.001
**Physical activity, mean (SD)**							
Total activity (MET-h/week)	28.17 (67.00)	21.38 (65.91)	0.81	60.85 (101.41)	52.28 (94.43)	<0.001	<0.001
Vigorous activity (MET-h/week)	11.36 (41.45)	8.11 (43.99)	0.93	29.53 (71.50)	24.93 (68.79)	<0.001	<0.001
Moderate activity (MET-h/week)	12.85 (29.27)	11.06 (28.41)	0.94	24.39 (42.80)	22.36 (41.49)	<0.001	<0.001
Sedimentary activity (h/week)	6.32 (3.26)	7.24 (3.69)	0.002	5.62 (3.32)	6.08 (3.48)	<0.001	<0.001
Walk or bicycle (MET-h/week)	3.97 (16.42)	2.21 (13.51)	0.68	6.93 (21.51)	4.99 (17.92)	<0.001	<0.001
**Complete blood count—whole blood, mean (SD)**							
Hemoglobin (g/dL)	13.34 (1.80)	13.61 (1.65)	0.121	14.12 (1.50)	14.00 (1.57)	<0.001	<0.001
Hematocrit (%)	39.31 (5.02)	40.25 (4.61)	0.028	41.32 (4.21)	41.13 (4.30)	0.009	<0.001

* *P-value was analysis between four groups*.

*BMI, body mass index; SBP, systolic blood pressure; DBP, diastolic blood pressure; MET, metabolic equivalent of task; SD, standard deviation*.

## Discussion

There are two major findings in the present study. First, compared with non-CHF participants, CHF participants exhibited higher BMI with lower energy and macronutrient intake, lower physical activity level, and longer rest time and hemodilution (lower hematocrit and hemoglobin). Second, higher water intake, sedentary time, and hematocrit were observed in CHF participants with BMI ≥ 30 kg/m^2^s, while energy and macronutrient intake and physical level were similar between CHF participants with BMI ≥ 30 kg/m^2^ and <30 kg/m^2^.

Excess body weight and a sedentary lifestyle are major public health problems worldwide (16). Obesity (BMI ≥ 30 kg/m^2^) has been considered as a major independent risk factor for cardiovascular disease (17). A previous study showed that up to 40% of patients with CHF suffered from obesity (8). The interplay between obesity and CHF is complex. Despite that obesity increases the risk of CHF, studies found that CHF patients who developed obesity or overweight (25–29.9 kg/m^2^) are associated with better prognosis compared with those who did not (18–20), with reductions in cardiovascular disease mortality (19 and 40%, respectively) and all-cause mortality (16 and 33%, respectively) compared to heart failure patients with normal weight (BMI 20–24.9 kg/m^2^), whereas heart failure patients with BMI < 20 kg/m^2^ increased total and cardiovascular mortality (27 and 20%, respectively) (21). This phenomenon is termed as “obesity paradox,” and several potential reasons may account for it, such as earlier presentation, different etiology, greater metabolic reserves, protective cytokines, more tolerance of cardiac medications, different cardiorespiratory fitness level, and less cachexia (22). Based on previous studies, our study termed “obesity” as “BMI ≥ 30 kg/m^2^” to investigate the differences for increased BMI in CHF participants.

In our analysis, we recognized that CHF participants presented with higher BMI than non-CHF participants and a higher proportion of BMI ≥ 30 kg/m^2^ among CHF participants. Meanwhile, we also noticed an inadequate energy and macronutrient intake in CHF participants, seen as significantly lower energy, protein, carbohydrate, total sugar, total fat, cholesterol, and water intake. This implied that nutritional deficiency existed in CHF participants. Malnutrition is commonly prevalent in CHF, with a reported incidence of 7.5 and 57% in severe or moderate malnutrition, respectively (23). These patients are older and frail, with negative calorie and nitrogen balance and energy availability (24) for physical activity, and are triggered by multiple factors such as anorexia, malabsorption secondary to intestinal edema, high energy demand, and cytokine-induced hypercatabolism (7, 25) and worse outcomes (6, 26, 27). However, when compared with CHF participants with BMI < 30 kg/m^2^, participants with BMI ≥ 30 kg/m^2^ did not show a distinct increase in energy and macronutrient intake. A constellation of researches revealed that failing myocardium utilized glucose, switching from fatty acids, as the primary energetic substrate to produce ATP (28), which may exert a protective role in preventing cardiomyocytes from oxidative radical excess and cell damage (29, 30) but a lower energy production (31). With more total fat intake and less glucose intake, cells may have the less energetic substrate to produce enough energy. This would affect the metabolism in the body and cause adipose tissue accumulation and subsequently increased body weight. This may imply that inadequate or unbalanced energy and macronutrient intake accounts for the increase in BMI or obesity among CHF participants. A daily caloric intake of about 29 kcal/kg and a daily protein intake of 1.2–1.4 g/kg were recommended for CHF patients at normal weights and a less energy intake was required (20–24 kcal/kg/day), and a reduction in dietary fat intake to about 25% of total caloric intake (0.6–0.8 g/kg/day) was required for overweight and obese CHF patients (32, 33).

Our study found lower hematocrit and hemoglobin levels in CHF participants, while CHF with BMI ≥ 30 kg/m^2^ participants had higher hematocrit and hemoglobin concentration than those with BMI < 30 kg/m^2^. These results demonstrated that hemodilution and fluid retention are common in CHF, especially in those with lower BMI level (BMI < 30 kg/m^2^). This observation may be another potential reason to explain the “obesity paradox.” Considerable evidence demonstrated that total blood volume and cardiac output are positively correlated with the degree of excess body weight (18, 22, 34) and further preserved or even increased skeletal muscle mass (i.e., lean mass) (35, 36), which may cause BMI to be higher, and vice versa. This may explain the phenomenon of elevated hematocrit and hemoglobin concentration in CHF with BMI ≥ 30 kg/m^2^ participants, and fluid retention may not be the reason for the increased BMI or obesity in CHF participants. Hemodilution was common in chronic heart failure (37) and has a deleterious effect as it may impair peripheral oxygen delivery (38) and is often neglected, as compensatory mechanisms may mask signs of volume (39). Our study found lower hematocrit and hemoglobin level in CHF participants, while CHF with BMI ≥ 30 kg/m^2^ participants had higher hematocrit and hemoglobin concentration than BMI < 30 kg/m^2^ ones. These results demonstrated that hemodilution and fluid retention are common in CHF, especially in those with lower BMI level (BMI < 30 kg/m^2^). Previous studies indicated a higher mortality rate in patients with hemodilution than in those with hemoconcentration in acute heart failure patients (15), while fluid restriction could only improve signs and symptoms of chronic heart failure in patients in moderate to severe chronic heart failure (40) and aggressive fluid removal positively affected survival (41). In the present study, CHF with BMI ≥ 30 kg/m^2^ participants had higher hematocrit and hemoglobin concentration than BMI < 30 kg/m^2^ ones, indicating that obese CHF participants were less likely to develop hemodilution. This could be one explanation for the “obesity paradox.” Furthermore, hemodynamic changes including increased stroke volume and increased arterial pressure may compensate the impaired peripheral oxygen delivery, leading CHF patients with increased BMI or obesity to have better prognoses. Still, we could not ignore that there was fluid retention in our CHF group, which implied an inadequate usage of diuretics and fluid management should be enhanced.

Another finding in our analysis is that we demonstrated that CHF participants had lower physical activity levels and spent a longer time in rest, especially participants with BMI ≥ 30 kg/m^2^. It was revealed that sedentary time caused metabolic alterations at the muscle level and next influenced gross metabolic disturbances in the whole body (42). It has been reported that prolonged sedentary time would impair mitochondrial function by elevating oxidative stress levels (43), which decreased the mitochondrial respiration level (44) and caused insufficient ATP production for daily activity and metabolism. Emerging evidences have demonstrated a significant dose–response association between sitting time and cardiovascular disease mortality (45–48), and the relative risks associated with sedentary time were higher among participants without regular physical activity (49, 50). Meanwhile, there have been proven cardioprotective effects of regular physical activity on cardiovascular health, improving cardiac compliance, reducing arterial stiffness and ventricular afterload, and finally reducing the risk of future cardiac dysfunction and improving cardiovascular outcomes (42). Moreover, researches showed that an increasing lean mass with resistance exercise training could effectively improve muscular fitness in CHF (51, 52), which could prevent sarcopenia (53) or even cachexia in CHF patients and have better prognosis (54).

Our investigation has several limitations. We only included macronutrient in our analysis and lacked the data of micronutrient intake. Therefore, the present study may have underestimated the nutritional deficit and its effects on obesity among participants. Also, details regarding the etiology, subtype (HF with reduced ejection fraction vs. HF with preserved ejection fraction), severity of HF, and complications were not available. Moreover, CHF participants included in our analysis were self-reported in the NHANES survey. This may lead to possible selection, reporting, and recall bias. Additionally, we did not include socioeconomic data, and thus we may neglect the socioeconomic impacts on nutrition intake and lifestyle self-management of CHF participants. Finally, we lacked follow-up data of these participants, including BMI changes and relative outcomes, so we were unable to recognize the effects of nutrients intake and physical activity on BMI changes and prognoses in participants with CHF.

## Conclusion

Higher water intake, sedentary time, and hematocrit were observed in BMI ≥ 30 kg/m^2^ CHF participants. It seems that longer resting time and an unbalanced diet may be associated with a higher prevalence of increased BMI or obesity in CHF participants. Future research is warranted to explore the mechanisms underlying this finding and whether intentional weight loss with combination of diet, exercise, and others could be contributing to better health outcomes in BMI ≥ 30 kg/m^2^ CHF participants.

## Data Availability Statement

The original contributions presented in the study are included in the article/Supplementary Material, further inquiries can be directed to the corresponding author/s.

## Ethics Statement

This study was reviewed and approved by the National Center for Health Statistics research ethics review board, and written informed consent was obtained from all NHANES participants.

## Author Contributions

TX: study concept and design, acquisition, analysis, or interpretation of data, and drafting of the manuscript. HZho: study concept and design, acquisition, analysis, or interpretation of data, and statistical analysis. ZM and HZha: acquisition, analysis, or interpretation of data, and statistical analysis. QZ and DX: critical revision of the manuscript for important intellectual content, administrative, technical, or material support, and study supervision. YZ and JZ: study concept and design, critical revision of the manuscript for important intellectual content, administrative, technical, or material support, and study supervision. All authors read and approved the final manuscript.

## Conflict of Interest

The authors declare that the research was conducted in the absence of any commercial or financial relationships that could be construed as a potential conflict of interest.
