# Supplementary material for: Congestive Heart Failure Exhibited Higher BMI With Lower Energy Intake and Lower Physical Activity Level: Data From the National Health and Examination Nutrition Survey
Source: Front Cardiovasc Med. 2021 Jun 9;8:680371. doi: 10.3389/fcvm.2021.680371 (PMC8221290; doi:10.3389/fcvm.2021.680371)
Supplement: Supplementary file 1 [file Data_Sheet_1.doc]

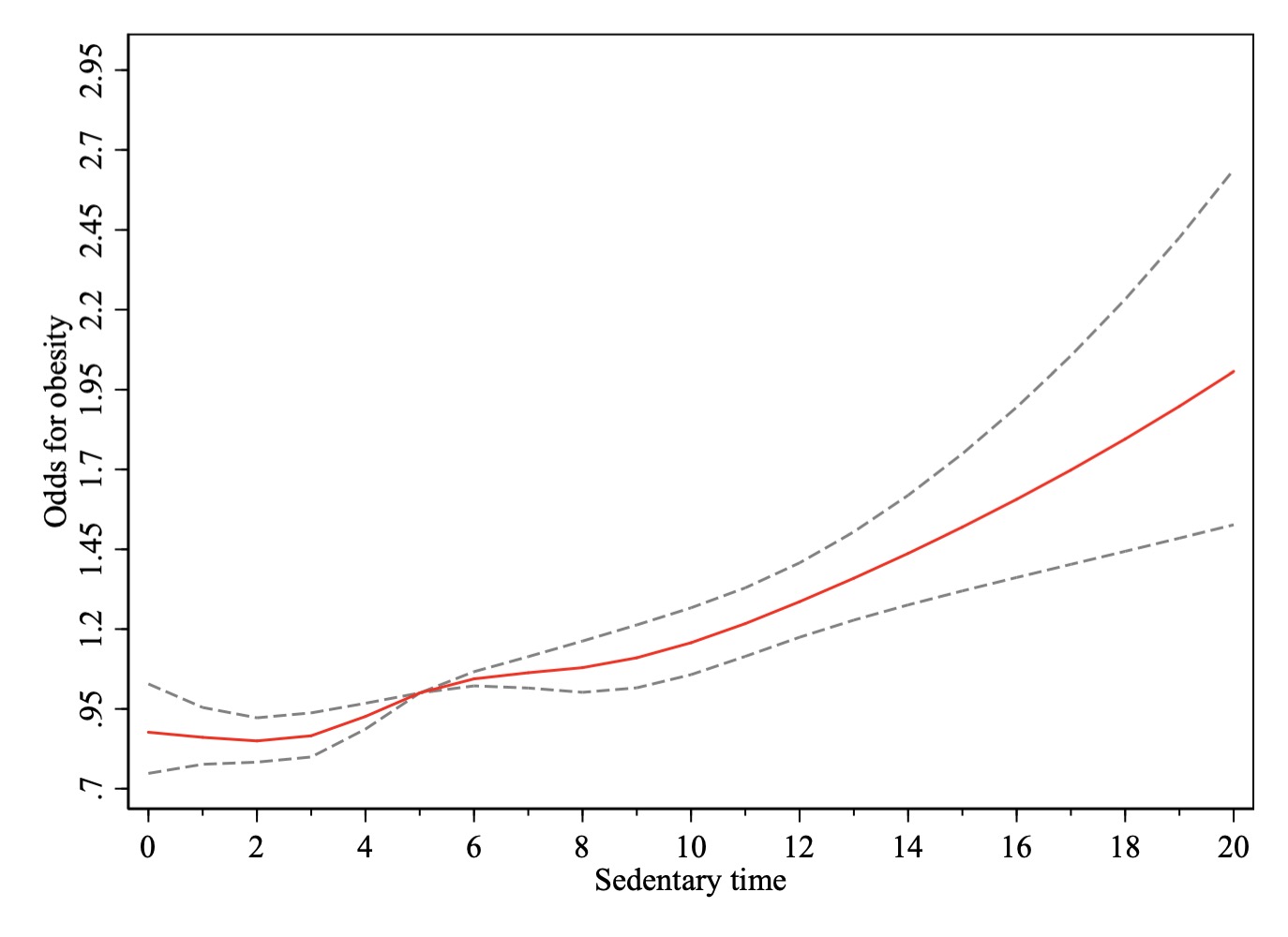


Odds ratio for BMI ≥ 30 kg/m^2^

Sedimentary time (h)

P for non-linearity 3.59

**Supplementary Figure 1.** Association of sedimentary time with BMI ≥ 30 kg/m^2^ in non-CHF participants. [Odds ratio 1.04,95% confidence interval(1.03-1.05)]
